# Case Report: Personalized, functional drug sensitivity-guided chemotherapy achieves long-term disease-free survival in canine pulmonary adenocarcinoma

**DOI:** 10.3389/fvets.2025.1678271

**Published:** 2026-01-16

**Authors:** Kyu-Duk Yeon, Kieun Bae, Jin-Young Choi, Kyong-Ah Yoon, Jung-Hyun Kim

**Affiliations:** 1 Department of Veterinary Internal Medicine, College of Veterinary Medicine, Konkuk University, Seoul, Republic of Korea; 2 SNC Animal Medical Center, Seoul, Republic of Korea; 3 KU Animal Cancer Center, Konkuk University Veterinary Medical Teaching Hospital, Seoul, Republic of Korea; 4 Department of Veterinary Biochemistry, College of Veterinary Medicine, Konkuk University, Seoul, Republic of Korea

**Keywords:** canine pulmonary adenocarcinoma, case report, drug sensitivity testing, functional precision oncology, organoid culture, personalized chemotherapy, targeted therapy

## Abstract

**Introduction:**

Canine pulmonary adenocarcinoma (PAC) is a relatively uncommon primary lung tumor in dogs, with prognosis influenced by clinical stage, histological grade, and surgical margins. Despite surgical resection being the treatment of choice, long-term outcomes remain highly variable, and the benefit of conventional empirically chosen adjuvant chemotherapy remains limited, especially in high-risk cases.

**Methods:**

A 10-year-old spayed female Maltese dog presented with a solitary pulmonary mass was diagnosed with moderately differentiated PAC after complete (R0) resection via right middle lung lobectomy. Given the tumor’s histological grade and suspected nodal involvement, *ex vivo* functional drug sensitivity testing using patient-derived tumor cells and three-dimensional organoid culture was performed to guide personalized chemotherapeutic selection.

**Results:**

Doxorubicin and toceranib exhibited the highest cytotoxicity and were sequentially administered as adjuvant therapy. The patient tolerated the treatment well without notable adverse effects, and serial thoracic imaging over 548 days revealed no evidence of recurrence or metastasis.

**Conclusion:**

This case highlights the clinical utility of integrating functional drug sensitivity testing and organoid validation into personalized chemotherapy decision-making for canine PAC, demonstrating prolonged disease-free survival exceeding 500 days in a patient with intermediate-grade histology and suspected nodal involvement.

## 1 Introduction

Primary pulmonary carcinoma (PPC) is a relatively uncommon malignancy in dogs, with pulmonary adenocarcinoma (PAC) being the most frequently reported histologic subtype (1). Although surgical excision is the treatment of choice, long-term outcomes remain highly variable and are strongly influenced by several prognostic factors. Specifically, tumor stage, histologic grade, completeness of surgical excision, and lymph node metastasis have been shown to significantly impact survival in canine PAC (1–3). Among these, the presence of lymph node involvement (N1) or a high histologic grade has consistently been associated with shorter disease-free intervals and overall survival times (2, 3).

Although adjuvant chemotherapy is often considered for dogs with high-risk features such as lymph node involvement or a moderate to high histologic grade, robust evidence for survival benefit remains limited. Traditional treatment decisions have relied heavily on clinical staging and histopathologic classification, which, while informative, may not fully capture the biological heterogeneity or chemosensitivity of individual tumors (1–3). This limitation underscores the need for additional strategies that can guide more personalized treatment selection beyond conventional prognostic parameters.

In human oncology, functional precision medicine has emerged as a promising approach to address this gap by tailoring therapy based on the direct measurement of drug response in patient-derived tumor cells (4). Drug sensitivity testing with patient-derived cells allows clinicians to identify agents with demonstrated *ex vivo* efficacy, and its predictive value is further enhanced when combined with three-dimensional (3D) organoid cultures. Compared to traditional 2D monolayers, organoid systems better preserve tumor architecture, cellular diversity, and microenvironmental features, offering a more physiologically relevant platform for assessing therapeutic responsiveness (5).

Despite its promise, functional precision oncology has seen limited application in veterinary medicine. However, its integration into clinical decision-making may offer substantial benefits, particularly for solid tumors with historically poor responsiveness to empiric chemotherapy protocols.

This report describes a case of canine pulmonary adenocarcinoma characterized by a moderately differentiated (grade II) tumor and suspected nodal involvement, for which adjuvant chemotherapy was considered based on histologic criteria. Rather than relying solely on standard protocols, *ex vivo* drug sensitivity testing and organoid validation were employed to guide personalized drug selection. To address these limitations, functional precision oncology offers a promising strategy by directly assessing drug sensitivity in patient-derived tumor cells. Despite increasing interest in human oncology, its application in veterinary medicine remains underexplored. Therefore, this report aims to present the feasibility and potential clinical relevance of integrating *ex vivo* drug sensitivity testing and three-dimensional organoid validation into individualized treatment planning for canine pulmonary adenocarcinoma. This case highlights the potential clinical utility of functional precision oncology in veterinary medicine.

## 2 Case description

### 2.1 Diagnostic assessment

**Figure 1:**
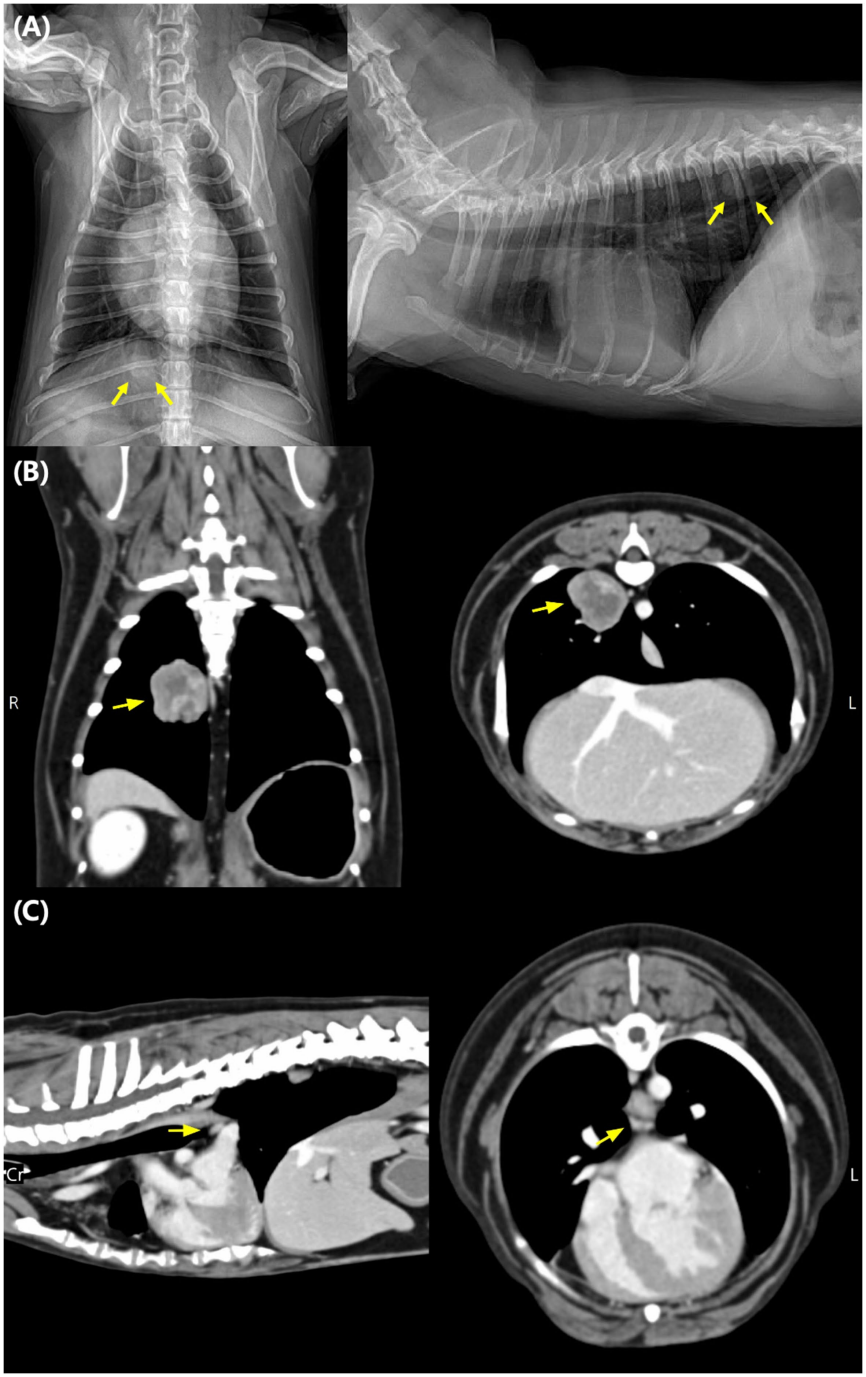
Thoracic imaging of a 10-year-old spayed female Maltese dog with a pulmonary mass. **(A)** Ventrodorsal and right thoracic radiographs showing a solitary, well-defined soft tissue opacity (yellow arrows) in the mid-right hemithorax, consistent with a pulmonary mass potentially arising from either the right middle lung lobe or the right caudal lung lobe. **(B)** Contrast-enhanced computed tomography images (coronal and transverse planes) demonstrating a heterogeneously enhancing mass (yellow arrows) within the right middle lung lobe. **(C)** Contrast-enhanced CT images (sagittal and transverse planes) confirming mild enlargement of the tracheobronchial lymph node adjacent to the carina (yellow arrows), which served as the imaging basis for presumptive N1 classification in this case.

A 10-year-old spayed female Maltese dog was referred following the incidental detection of a solitary pulmonary mass during a routine geriatric health screening. On ventrodorsal and right lateral thoracic radiographs, a solitary, well-defined soft-tissue opacity was observed in the mid-right hemithorax, consistent with a pulmonary mass potentially originating from either the right middle lung lobe or the right caudal lung lobe. No pleural effusion or additional pulmonary lesions were identified (Figure 1A). Thoracic computed tomography (CT) revealed a heterogeneously enhancing mass measuring 20.9 × 20.9 × 22.6 mm within the right middle lung lobe. The mass was closely associated with adjacent bronchial structures. Mild enlargement of the tracheobronchial lymph node adjacent to the carina was confirmed on contrast-enhanced CT (Figure 1C), providing the imaging basis for presumptive N1 staging noted in the original submission. However, cytologic or histologic confirmation was not feasible due to the node’s deep anatomic location, and therefore the N1 classification remains presumptive rather than pathologically confirmed. For comprehensive staging, whole-body CT (thoracic–abdominal) and abdominal ultrasonography were performed, which did not identify any abnormalities suggestive of distant metastasis. Prior to administering doxorubicin, a complete echocardiographic examination confirmed normal systolic function with no structural cardiac abnormalities. These findings are illustrated in Figure 1, which depicts the pulmonary mass on thoracic radiographs (A), contrast-enhanced CT scans of the mass (B), and mild tracheobronchial lymphadenopathy (C).

**Figure 2:**
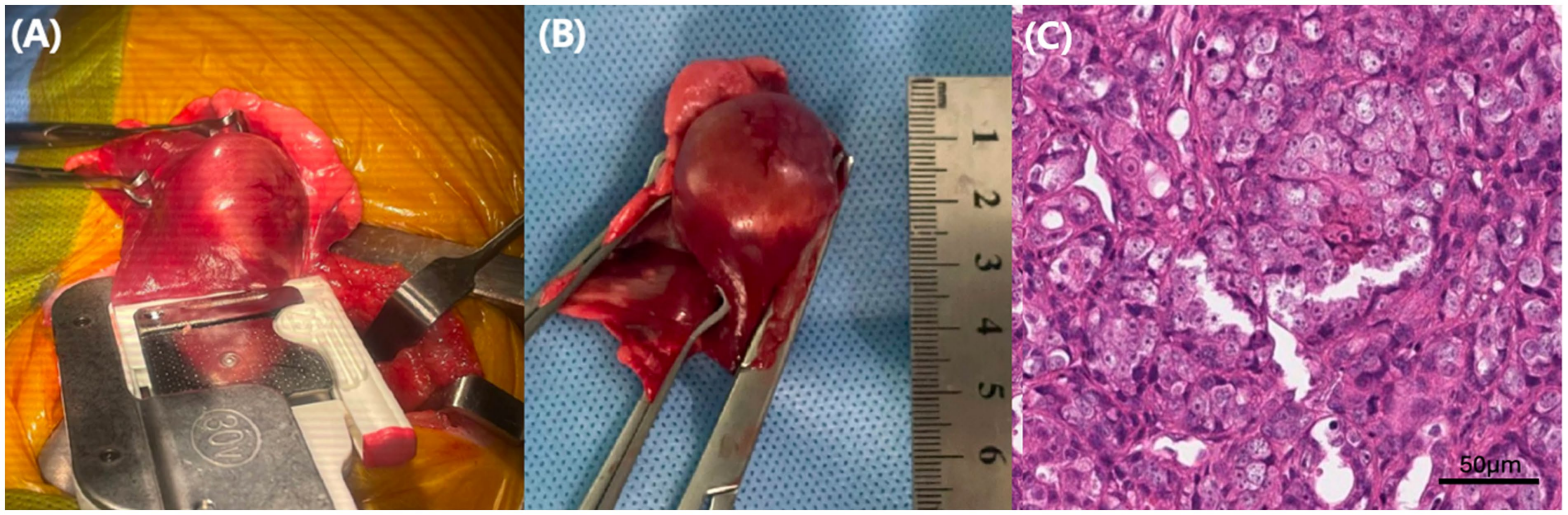
Surgical and histopathological features of the resected pulmonary mass. **(A)** Intraoperative image obtained during right middle lung lobectomy using a thoracoabdominal stapler, demonstrating isolation of the pulmonary mass. **(B)** Gross appearance of the excised right middle lung lobe, showing a well-circumscribed, firm mass. **(C)** Histopathologic section of the tumor (H&E stain, 20×; scale bar = 50 μm), showing papillary, tubular, and focally solid growth patterns, with moderate nuclear pleomorphism, a mitotic index of 11–20 per 10 high-power fields, and 21–50% tumor necrosis—findings consistent with a moderately differentiated (grade II) pulmonary adenocarcinoma.

On day 0, a right middle lung lobectomy was perfomed via lateral thoracotomy. A thoracoabdominal stapling device was used to transect the bronchovascular pedicle and excise the affected lung lobe. Histopathological examination confirmed a moderately differentiated pulmonary adenocarcinoma (Grade II), exhibiting papillary and tubular structures with an invasive growth pattern. The mitotic index was 11–20 per 10 high-power fields, accompanied by moderate nuclear pleomorphism and nucleolar prominence. Extensive necrosis involving 21–50% of the tumor area was observed, supporting a classification of Grade II. All surgical margins were evaluated and found to be free of tumor infiltration on H&E staining, with no neoplastic cells observed at the inked resection margins, consistent with complete (R0) resection. No evidence of vascular or lymphatic invasion was identified. The gross and microscopic appearances of the resected lung lobe are shown in Figure 2, highlighting the features of a moderately differentiated pulmonary adenocarcinoma.

**Figure 3:**
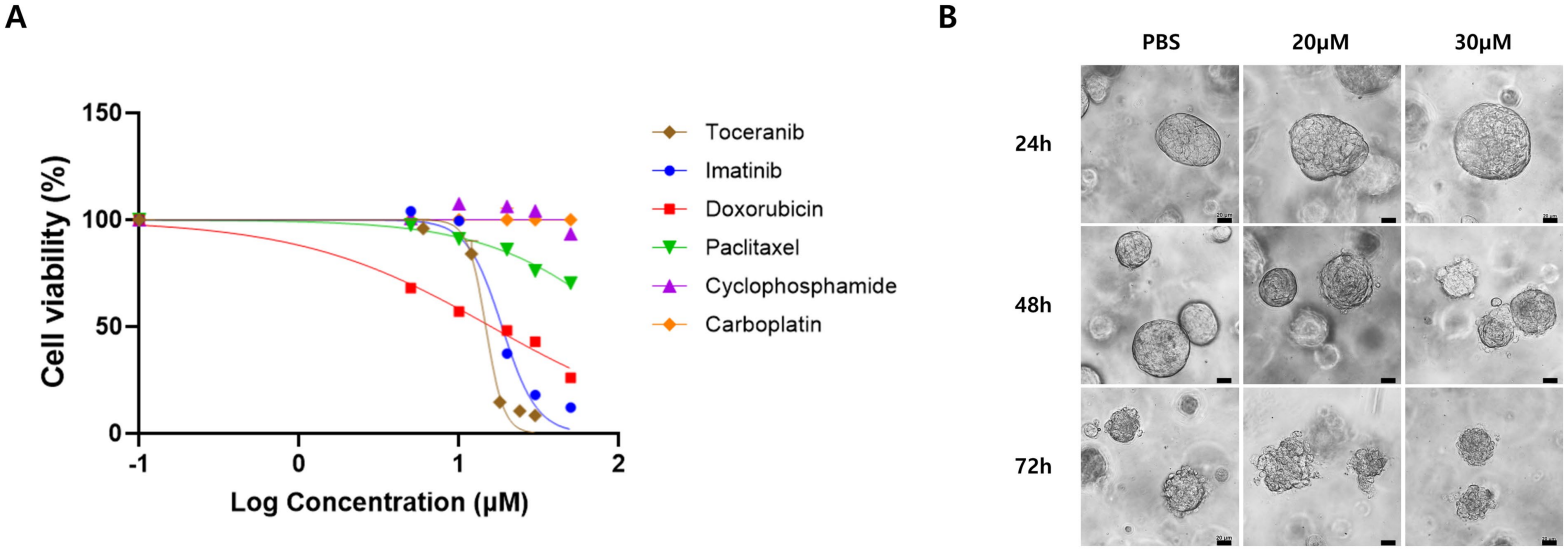
Drug sensitivity analysis of primary pulmonary adenocarcinoma cells. **(A)** Dose–response curves of cell viability (%) after 24-h exposure to six chemotherapeutic agents, assessed using the ATP-based cell viability assay. Toceranib and doxorubicin exhibited the most potent cytotoxicity among the tested agents. **(B)** Phase-contrast images of 3D organoids derived from the same tumor, treated with toceranib at 20 or 30 μm. Organoids showed shrinkage and structural collapse in a time- (24, 48, and 72 h) and dose-dependent manner. Higher concentrations induced more rapid and pronounced organoid collapse. Scale bars = 20 μm.

Surgically resected tumor tissue was subjected to 2D and 3D organoid cultures. The tumor was mechanically minced and enzymatically dissociated using a gentleMACS™ Dissociator (Miltenyi Biotec, Germany). Isolated cells were cultured in Advanced DMEM/F12 medium supplemented with 10% fetal bovine serum, GlutaMAX™ and Zellshield at 37 °C in a 5% CO₂ atmosphere. For drug screening, cells were seeded in 96-well plates (1.5 × 10^4^ cells/well) and exposed to six chemotherapeutic agents–doxorubicin, toceranib, imatinib, paclitaxel, cyclophosphamide, and carboplatin–across concentrations ranging from 5 to 100 μM. The concentration range was selected based on previously published *in vitro* studies to assess dose-dependent cellular responses (6). After 24 h, cell viability was assessed using the CellTiter-Glo® luminescent assay. To quantitatively evaluate drug sensitivity, the half maximal inhibitory concentration (IC₅₀) values were calculated from dose–response curves generated by nonlinear regression analysis. Among the tested agents, doxorubicin and toceranib demonstrated the highest cytotoxicity (Supplementary Table S1; Figure 3A). For three-dimensional (3D) organoid culture, tumor-derived cells were embedded in Matrigel and maintained in Advanced DMEM/F12 containing A83-01, B27, N2, EGF, FGF10, R-spondin-1, Wnt3a and Zellshield, following established 3D organoid methodologies described in previous literature (7). Organoids were cultured for 5 days before drug treatment, and 3D organoids exhibited clear time- and dose-dependent collapse in response to toceranib exposure (Figure 3B), supporting the *in vitro* findings. Both 2D cultures and 3D organoids were used at passages 2–4, and organoids with >80% viability and consistent formation within 5–7 days were included in drug treatment.

### 2.2 Therapeutic intervention

Based on these findings, a personalized chemotherapy regimen was initiated. Doxorubicin and toceranib were selected due to their high cytotoxicity in the *in vitro* drug sensitivity assay, with IC₅₀ values of 15.98 ± 1.86 μM for doxorubicin and 14.78 ± 0.97 μM for toceranib (Supplementary Table S1; Figure 3A). These results indicated strong susceptibility and were further supported by time- and dose-dependent organoid collapse in 3D organoid cultures (Figure 3B). Intravenous doxorubicin was administered at 1 mg/kg every 3 weeks for a total of five cycles, starting on Day 33. The dosing protocol was selected based on previously established guidelines and literature supporting safety and efficacy in canine solid tumors, including Withrow and MacEwen’s Small Animal Clinical Oncology (6th ed.) (8).

Premedication included chlorpheniramine (0.5 mg/kg IM), omeprazole (0.7 mg/kg IV), and maropitant (1 mg/kg SC). The treatment was well tolerated, and only Grade 1 decreased appetite was observed according to the VCOG-CTCAE v2 criteria (9). Hematologic parameters, including CBC and serum biochemistry, were assessed at nearly every visit during the doxorubicin phase. For the first three chemotherapy cycles, additional follow-up visits were conducted approximately 7 days after each treatment to monitor for delayed toxicity. No hematologic or clinical adverse effects were observed. During toceranib maintenance therapy, the patient was re-evaluated every 3 to 4 weeks for physical examination, laboratory monitoring, and thoracic radiographs. Thoracic imaging was performed at each visit to monitor for recurrence, although only representative timepoints are presented in Supplementary Figure S1. No abnormalities were detected throughout the course of treatment (Supplementary Table S2).

### 2.3 Follow-up and outcomes

On day 140, maintenance therapy with oral toceranib therapy (2.75 mg/kg, three times weekly) was initiated. The patient remained clinically stable, with no gastrointestinal or systemic adverse effects noted. Serial thoracic radiographs and abdominal ultrasonography, performed every 2 to 3 months, showed no evidence of local recurrence or metastasis (Supplementary Figures S1, S2). Although follow-up CT was considered due to the initial suspicion of bronchial invasion, it was not performed because the patient remained asymptomatic with stable thoracic radiographs. Given the lack of clinical progression and upon discussion with the owner, advanced imaging was not elected.

At the time of writing, the patient remains alive 548 days after initial diagnosis, corresponding to a disease-free interval of 525 days following surgical resection (Day 0).

A timeline of the clinical course is summarized in Table 1.

**Table 1:** Timeline of major clinical events including diagnosis, chemotherapy, and follow-up evaluations.

Day	Clinical event
D0	Surgical lobectomy (R0 resection)
D33	Doxorubicin #1 (1 mg/kg IV)
D54	Doxorubicin #2
D75	Doxorubicin #3
D96	Doxorubicin #4
D117	Doxorubicin #5 (end of IV chemo)
D140	Oral toceranib started (2.75 mg/kg, 3×/week)
D183	Toceranib (1 month follow-up)
D243	Toceranib (3 months)
D393	Toceranib (6 months)
D586	Final follow-up (alive, disease-free)

## 3 Discussion

The prognosis of canine pulmonary adenocarcinoma is primarily influenced by clinical stage and histologic grade, both of which guide treatment decisions and expected outcomes (2). The presence of lymph node metastasis is associated with a marked reduction in survival, with median survival times (MSTs) ranging from 60 to 167 days in lymph node-positive (LN+) dogs, compared to MSTs exceeding 400 days in LN-negative cases (3). Histologic grade has also been variably associated with prognosis. For example, McNiel et al. (2) reported MSTs of 545, 211, and 61 days for grade I, II, and III tumors, respectively, demonstrating an inverse relationship between grade and survival (2). Although this study predates the widespread use of advanced imaging and adjuvant chemotherapy, it remains one of the few to stratify survival by histologic grade. Conversely, a more recent study by Ichimata et al. (1) in small-breed dogs found no statistically significant difference in survival between grade I and II tumors, suggesting that the prognostic value of histologic grade may be context-dependent (1).

In the present case, thoracic CT suggested T1N1M0 disease, although pathologic confirmation of lymph node involvement was not obtained because tracheobronchial lymph node biopsy or excision was not performed as part of the planned surgical procedure, following intraoperative assessment and risk–benefit considerations. Histopathologic examination confirmed a moderately differentiated (grade II) pulmonary adenocarcinoma, which is generally considered intermediate-grade and associated with increased risk of recurrence and reduced survival (2). Based on these findings, adjuvant chemotherapy was considered appropriate.

It should be noted, however, that the tracheobronchial lymphadenopathy in this case was identified on contrast-enhanced CT (Figure 1C) but was not histologically confirmed. Therefore, the clinical staging of T1N1M0 should be interpreted with caution, as the disease might have represented an early-stage (T1N0M0) pulmonary adenocarcinoma rather than true nodal metastasis. This aligns with recent literature suggesting that mild tracheobronchial lymphadenopathy on imaging alone is insufficient to confirm nodal involvement and may lead to presumptive, rather than definitive, N classification. This uncertainty may influence the interpretation of prognosis, because early-stage tumors in dogs can achieve long-term survival following surgery alone (10). As raised by external reviewers, such presumptive nodal staging may overestimate disease burden and complicate interpretation of adjuvant treatment necessity in otherwise early-stage pulmonary adenocarcinoma. Nevertheless, adjuvant therapy was pursued not solely because of suspected nodal involvement but also to explore the feasibility of applying functional precision oncology using *ex vivo* drug sensitivity testing and organoid validation.

It is also important to note that even if this case were reclassified as early-stage disease (T1N0M0), previous studies have shown no significant survival advantage from adjuvant chemotherapy following complete resection. Ichimata et al. (1) reported that dogs with early-stage pulmonary adenocarcinoma achieved a median survival time (MST) of 759 days with adjuvant chemotherapy and an unreached MST in dogs managed by surgery alone (*p* = 0.753), indicating that postoperative chemotherapy may not provide a clear benefit in such cases (1). Therefore, the long-term disease-free survival of 548 days observed in the present case falls within the expected survival range for early-stage tumors treated with surgery alone. However, adjuvant therapy in this case was not intended as a standard postoperative treatment but rather as a proof-of-concept application of functional precision oncology aimed at evaluating its clinical feasibility and translational potential.

Most previous reports of canine pulmonary adenocarcinoma either omitted adjuvant chemotherapy altogether or used standard agents selected empirically without functional testing (1–3). In the present case, however, a regimen was tailored to the tumor’s specific drug susceptibility profile, identifying doxorubicin and toceranib as the most effective agents. Despite achieving R0 resection and lacking histologic evidence of vascular or lymphatic invasion, the tumor exhibited features suggestive of aggressive behavior, including a mitotic index of 11–20 per 10 high-power fields, moderate nuclear pleomorphism, and 21–50% necrosis.

The long-term outcome observed in this patient, with a survival time of 548 days and a disease-free interval of 525 days, illustrates that functional precision oncology can be feasibly incorporated into the management of canine solid tumors but does not by itself demonstrate superiority over conventional treatment.

However, although doxorubicin and toceranib demonstrated the greatest ex vivo cytotoxicity in this assay system, the IC50 values clearly exceeded the reported therapeutic plasma concentrations in dogs. Therefore, these findings should not be interpreted as evidence of pharmacologic sensitivity *in vivo.* Instead, the DST results reflect relative phenotypic susceptibility under controlled *in vitro* conditions. The use of these agents in this case represents an exploratory application of functional precision oncology rather than a conventional, evidence-based adjuvant chemotherapy decision, as drug selection was based on relative phenotypic ranking within the ex vivo assay rather than on assumptions of clinically achievable systemic concentrations.

Toceranib, a multi-targeted receptor tyrosine kinase (RTK) inhibitor, inhibits VEGFR, PDGFR, and KIT, thereby suppressing angiogenesis, stromal support, and tumor proliferation (11).

This multi-faceted mechanism may be advantageous in canine pulmonary adenocarcinoma, where aberrant angiogenic and stromal signaling is suspected to contribute to tumor progression. In particular, recent molecular studies in canine lung tumors have shown upregulation of VEGFR and PDGFR expression, suggesting that these pathways may serve as relevant therapeutic targets in selected cases of PAC (3). Although molecular profiling was not performed in this case, the marked *ex vivo* sensitivity to toceranib observed in both 2D cell viability and 3D organoid assays implies that dysregulation of these receptor-mediated pathways could have contributed to the observed therapeutic responsiveness.

From a functional precision medicine perspective, this case illustrates how *ex vivo* drug-sensitivity testing can complement molecular data by providing direct, phenotype-level evidence of drug efficacy. Such approaches have recently gained traction in veterinary oncology, bridging the gap between empirical treatment and molecularly guided therapy (4, 12). For example, Fonseca-Alves et al. (12) and Wu et al. (13) reported that integrating functional assays with genomic insights can improve treatment selection and outcome prediction in dogs with various solid tumors (12, 13). These studies collectively support the translational value of functional precision oncology in veterinary medicine.

Doxorubicin, an anthracycline that intercalates DNA and inhibits topoisomerase II, induces apoptosis in rapidly proliferating tumor cells. When combined with an RTK inhibitor targeting VEGFR/PDGFR signaling, chemotherapy-induced cytotoxicity may be amplified through tumor microenvironment modulation and enhanced immune accessibility. This mechanistic synergy may explain the durable disease control achieved in this case despite intermediate-grade histology and presumptive nodal involvement.

Although *in vitro* drug sensitivity results do not always translate directly into clinical response due to tumor heterogeneity, microenvironmental complexity, and inter-patient variability, functional assays nonetheless provide actionable phenotypic data that can guide rational chemotherapy selection (4, 14). Limitations of this approach include the need for viable tumor tissue, technical expertise, and costs associated with 3D organoid culture systems (7). Nevertheless, when integrated with histologic and clinical findings, functional drug testing may enhance decision-making, even for solid tumors historically considered refractory to chemotherapy.

This case underscores the emerging potential of functional precision oncology to inform individualized chemotherapy protocols in canine solid tumors. As more studies incorporate *ex vivo* drug testing and multi-omic profiling, the integration of phenotypic and molecular evidence may establish a new paradigm for precision medicine in veterinary oncology (12, 13).

Future research should aim to standardize functional drug sensitivity testing protocols and evaluate their reproducibility across institutions. In particular, integrating functional assays with molecular and genomic profiling may offer a more comprehensive understanding of tumor biology, enabling the development of hybrid precision oncology platforms (15). Prospective multicenter studies incorporating *ex vivo* drug testing with next-generation sequencing may help establish robust, clinically actionable frameworks for personalized treatment in veterinary oncology (14, 16).

While this case provides promising insights into the potential of functional drug sensitivity testing to guide personalized chemotherapy for canine solid tumors, several important limitations must be acknowledged. First, this is a single-case report, and the findings may not be generalizable to broader populations. Second, because tracheobronchial lymphadenopathy was documented only on contrast-enhanced CT and not histologically confirmed, the clinical stage in this dog remains uncertain and may have represented early-stage (T1N0M0) disease rather than true nodal metastasis. In light of reports showing that some early-stage pulmonary adenocarcinomas achieve long-term control with surgery alone (1, 10), the favourable survival observed in this case cannot be interpreted as proof of benefit from the selected agents. Moreover, the cytotoxic concentrations observed *in vitro* exceeded physiologically attainable systemic exposure in dogs, so the ex vivo drug sensitivity testing results should not be regarded as validation of clinical therapeutic efficacy but rather as a relative ranking of drug susceptibility under experimental conditions.

Accordingly, the present report is best viewed as an exploratory proof-of-concept example of how functional drug testing and organoid validation may be incorporated into individualized treatment planning in veterinary oncology. Despite encouraging feasibility, technical variability, tumor heterogeneity, costs, and the lack of standardized protocols remain important barriers to broader clinical implementation of these methods. Prospective multicentre studies that integrate standardized functional drug sensitivity platforms with molecular profiling will be required to determine whether functional precision oncology can consistently improve outcomes compared with conventional management in dogs with pulmonary adenocarcinoma and other solid tumors.

## 4 Conclusion

This case demonstrates the potential utility of functional precision oncology in guiding individualized adjuvant therapy for canine pulmonary adenocarcinoma. By integrating *ex vivo* drug sensitivity testing and three-dimensional organoid validation, a tailored regimen was selected based on the tumor’s phenotypic drug response rather than empirical protocols. Although the long-term outcome cannot be attributed solely to the selected agents, the favorable disease control observed in this patient highlights the feasibility and translational relevance of incorporating functional assays into treatment planning. Continued investigation through standardized protocols and prospective studies is warranted to further define the clinical value of functional precision approaches in veterinary oncology.

## 5 Patient perspective

According to the owner, the patient remained bright and active throughout treatment. They expressed gratitude for the personalized approach and were satisfied with the outcome.

## Data Availability

The original contributions presented in the study are included in the article/Supplementary material, further inquiries can be directed to the corresponding author.

## Supplementary material

SUPPLEMENTARY FIGURE S1 Serial thoracic radiographs obtained during postoperative follow-up at **(A)** day 0, **(B)** 7 weeks (day 54), **(C)** 6 months (day 182), and **(D)** 17 months (day 525).

SUPPLEMENTARY FIGURE S2 Serial abdominal ultrasonographic images of the liver and spleen obtained during postoperative follow-up at **(A)** day 0, **(B)** 7 weeks (day 54), **(C)** 6 months (day 182), and **(D)** 17 months (day 525).
