# Supplementary figures and images for: Case Report: Personalized, functional drug sensitivity-guided chemotherapy achieves long-term disease-free survival in canine pulmonary adenocarcinoma
Source: Front Vet Sci. 2026 Jan 16;12:1678271. doi: 10.3389/fvets.2025.1678271 (PMC12856916; doi:10.3389/fvets.2025.1678271)

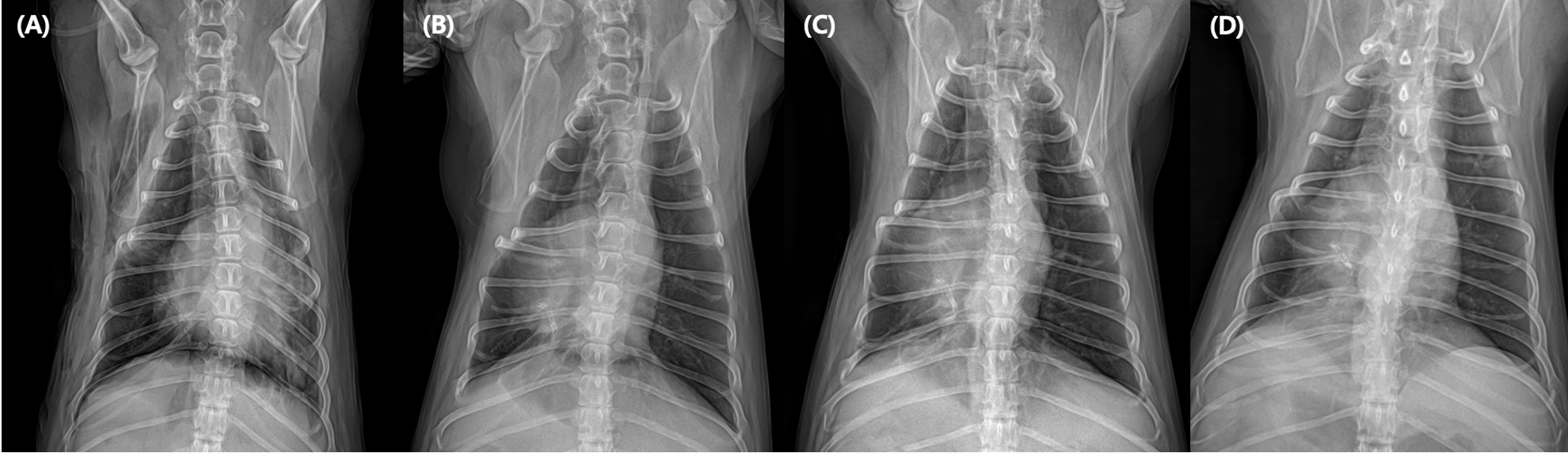

Supplement: SUPPLEMENTARY FIGURE S1 — Serial thoracic radiographs obtained during postoperative follow-up at (A) day 0, (B) 7 weeks (day 54), (C) 6 months (day 182), and (D) 17 months (day 525). [file Image_1.TIFF]

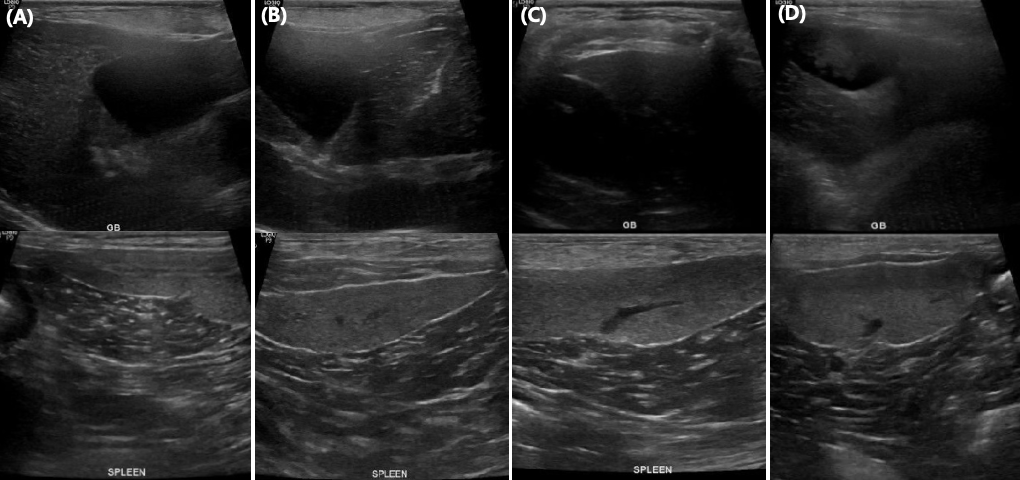

Supplement: SUPPLEMENTARY FIGURE S2 — Serial abdominal ultrasonographic images of the liver and spleen obtained during postoperative follow-up at (A) day 0, (B) 7 weeks (day 54), (C) 6 months (day 182), and (D) 17 months (day 525). [file Image_2.TIFF]
