# Supplementary material for: Case Report: Personalized, functional drug sensitivity-guided chemotherapy achieves long-term disease-free survival in canine pulmonary adenocarcinoma
Source: Front Vet Sci. 2026 Jan 16;12:1678271. doi: 10.3389/fvets.2025.1678271 (PMC12856916; doi:10.3389/fvets.2025.1678271)
Supplement: Supplementary file 3 [file Table_1.DOCX]

**Supplementary** **Table 1.** In vitro drug sensitivity results from cell viability assay using primary tumor cells.

| **Drug** | **Vehicle** | **Tested concentrations (μM)** | **IC50 (μM)** | **Estimated sensitivity*** |
| --- | --- | --- | --- | --- |
| Doxorubicin | PBS | 5, 10, 20, 30, 50 | 15.98 ± 1.86 | High |
| Toceranib | PBS | 6, 12, 18, 24, 30 | 14.78 ± 0.97 | High |
| Imatinib | DMSO | 5, 10, 20, 30, 50 | 17.76 ± 1.39 | High |
| Paclitaxel | DMSO | 5, 10, 25, 50, 100 | > 100 | Resistant |
| Cyclophosphamide | DMSO | 5, 10, 25, 50, 100 | > 100 | Resistant |
| Carboplatin | PBS | 5, 10, 25, 50, 100 | > 100 | Resistant |

*Sensitivity interpretation was defined based on IC50 thresholds: High (<30 μM), Moderate (30–60 μM), Low (60–100 μM), Resistant (>100 μM).**Supplementary** **Table 2.** Serial hematologic and biochemical monitoring during and after doxorubicin chemotherapy.

| **Day** | **Treatment** | **WBC (×10³/μL)** | **HCT (%)** | **PLT (×10³/μL)** | **ALT (U/L)** | **BUN (mg/dL)** | **Adverse Events** | **BW (kg)** | **Notes** |
| --- | --- | --- | --- | --- | --- | --- | --- | --- | --- |
| D33 | Doxorubicin #1 | 6.3 | 43.5 | 514 | 71 | 18 | None | 5.5 | Baseline before chemotherapy |
| D54 | Doxorubicin #2 | 8.32 | 42.7 | 452 | 132 | 26 | None | 5.6 | — |
| D75 | Doxorubicin #3 | 5.82 | 47.7 | 489 | 64 | 30 | None | 5.6 | — |
| D96 | Doxorubicin #4 | 6.9 | 44.0 | 433 | 69 | 25 | None | 5.6 | — |
| D117 | Doxorubicin #5 | 5.98 | 41.7 | 485 | 82 | 24 | None | 5.6 | End of IV chemo |
| D153 | Toceranib started | 6.73 | 40.5 | 513 | 90 | 30 | None | 5.5 | Oral maintenance begins |
| D183 | Toceranib (1 month) | 6.44 | 46.1 | 445 | 96 | 35 | None | 5.5 | — |
| D243 | Toceranib (3 months) | 5.17 | 42.9 | 416 | 63 | 37 | None | 5.6 | — |
| D393 | Toceranib (6 months) | 4.2 | 38.7 | 436 | 87 | 33 | None | 5.7 | — |
| D586 | Final follow-up | 5.28 | 38.3 | 526 | 78 | 35 | None | 5.5 | No recurrence or metastasis |

All values remained within acceptable ranges, and no adverse clinical signs were observed during or after chemotherapy.

**Abbreviations:** WBC, white blood cell count; HCT, hematocrit; PLT, platelet count; ALT, alanine aminotransferase; BUN, blood urea nitrogen; BW, body weight; IV, intravenous
